# Enterobacterial Common Antigen: Synthesis and Function of an Enigmatic Molecule

**DOI:** 10.1128/mBio.01914-20

**Published:** 2020-08-11

**Authors:** Ashutosh K. Rai, Angela M. Mitchell

**Affiliations:** a Department of Biology, Texas A&M University, College Station, Texas, USA; University of Texas Health Science Center at Houston

**Keywords:** O-antigen, cross-reactivity, enterobacterial common antigen, outer membrane, surface antigens

## Abstract

The outer membrane (OM) of Gram-negative bacteria poses a barrier to antibiotic entry due to its high impermeability. Thus, there is an urgent need to study the function and biogenesis of the OM. In *Enterobacterales*, an order of bacteria with many pathogenic members, one of the components of the OM is enterobacterial common antigen (ECA). We have known of the presence of ECA on the cell surface of *Enterobacterales* for many years, but its properties have only more recently begun to be unraveled. ECA is a carbohydrate antigen built of repeating units of three amino sugars, the structure of which is conserved throughout *Enterobacterales.* There are three forms of ECA, two of which (ECA_PG_ and ECA_LPS_) are located on the cell surface, while one (ECA_CYC_) is located in the periplasm. Awareness of the importance of ECA has increased due to studies of its function that show it plays a vital role in bacterial physiology and interaction with the environment. Here, we review the discovery of ECA, the pathways for the biosynthesis of ECA, and the interactions of its various forms. In addition, we consider the role of ECA in the host immune response, as well as its potential roles in host-pathogen interaction. Furthermore, we explore recent work that offers insights into the cellular function of ECA. This review provides a glimpse of the biological significance of this enigmatic molecule.

## Abstract (precis)

The outer membrane (OM) of Gram-negative bacteria poses a barrier to antibiotic entry due to its high impermeability. Thus, there is an urgent need to study the function and biogenesis of the OM. In *Enterobacterales*, an order of bacteria with many pathogenic members, one of the components of the OM is enterobacterial common antigen (ECA). We have known of the presence of ECA on the cell surface of *Enterobacterales* for many years, but its properties have only more recently begun to be unraveled.

## INTRODUCTION

Diverse environmental conditions on Earth (e.g., heat, pH, salinity, pressure, and osmotic activity) immensely affect the function of the cell, necessitating adaptation through structural modification. Gram-negative bacteria have an impermeable and strengthened outer membrane (OM) that allows them to withstand stress brought about by environmental factors, including other bacteria, antibiotics, and chemical stresses. The cell envelope structure of Gram-negative bacteria consists of the inner membrane, the periplasm containing the peptidoglycan cell wall, and the OM (1). The lipids of the OM form a barrier that is impermeable to large hydrophilic and hydrophobic molecules (2). Lipopolysaccharide (LPS) facilitates the formation of this barrier though (i) the high number of fatty acyl substituents per lipid molecule, which form a gel-like structure enhancing the rigidity of membrane (3, 4), (ii) strong lateral interaction between LPS molecules mediated by salt bridges with divalent cations (5), and (iii) modification of LPS structure in response to different environmental conditions (6). For example, in *Salmonella*, the PhoPQ two-component system causes antimicrobial peptide resistance after induction by divalent cation starvation by activating PagP (7). PagP facilitates the addition of palmitate chain to lipid A, altering the fluidity of the LPS molecules in the OM (7).

OM proteins (OMPs) are integral membrane proteins present in the membranes of Gram-negative bacteria, mitochondria, and chloroplasts. These proteins adopt a β-barrel architecture arranged in the membrane in anti-parallel patterns (8). Some of these proteins (porins) can form pores in the OM (2). These OMPs regulate the movement of small hydrophilic molecules across the outer membrane, such as nutrients, water, ions, and some small hydrophilic antibiotics (2). In fact, in Escherichia coli, β-lactam antibiotics, tetracyclines, chloramphenicol, and fluoroquinolones quickly diffuse through OmpF (9–12). Specific porins can also transport amphipathic substrates. For instance, transportation of long-chain fatty acid is facilitated by the lipid transporter FadL (13). Beyond its role controlling the entry of molecules into the cell, the OM plays a structural role, providing protection against mechanical and osmotic stresses (14, 15).

**FIG 1:**
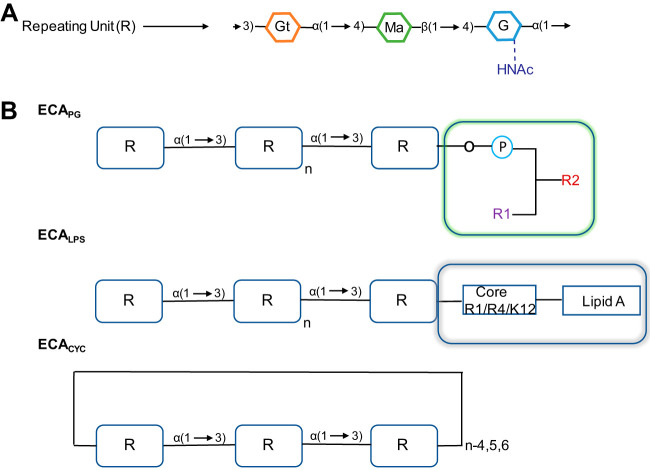
The structure of ECA. (A) The structure of the repeating unit (R) of ECA is made up of amino sugars (G, *N*-acetylglucosamine; Ma; *N*-acetyl-d-mannosaminuronic acid; Gt, 4-acetamido-4,6-dideoxy-d-galactose). (B) Structural differences between the three ECA forms. ECA_PG_, phospholipid-linked ECA; ECA_LPS_, lipopolysaccharide-linked ECA; ECA_CYC_, cyclic form of ECA. In the ECA_PG_ structure, R1 (-CH_2_OH group), and R2 (-CHOH group) indicate acyl chains. In the ECA_LPS_ structure, “core” represents the core polysaccharide of LPS, which is attached to lipid A, a hydrophobic lipid section that anchors LPS to the outer membrane. In the core region, the common tetrasaccharide structure is substituted for R1 (β-glucose) and R4 (β-galactose) compared to the K-12 core. n, a variable number of ECA repeating units. ECA_CYC_ generally consists of 4 to 6 repeating units (R) depending on the species. For example, in E. coli, 4 repeating units are present.

The Gram-negative OM is coated in highly variable molecules that can cause immune activation, known as antigens. Bacteria are divided into serotypes based on different antigen combinations (16). The three major types of antigens present on the cell surface are O (somatic), K (capsular), and H (flagellar) (17, 18). These antigens can play roles in motility (H-antigen), protection from a hostile environment (K-antigens and O-antigen), interaction with the environment (K-antigens and O-antigen), and increasing the ability of the OM to provide structural support to the cell (O-antigen) (15, 19–21). The outer leaflet of the OM is made mainly of lipopolysaccharide (LPS), which consists of lipid-A, the core polysaccharide, and O-antigen (1). O-antigen is a highly variable chain of carbohydrates and thus is serotype specific. K-antigens are the capsule, a coat on the surface of bacteria outside the cell envelope. They generally consist of high-molecular-weight polysaccharides with some exceptions (e.g., K-88 and K-99 of E. coli, which are protein antigens) (22–24). The H-antigen is a protein antigen based on flagellar structure (25). *Enterobacterales* is a bacterial order that is defined in part by the presence of an antigen known as enterobacterial common antigen (ECA) (26). ECA, a carbohydrate antigen, is located in the outer leaflet of the OM and in the periplasm (27–30). Although *Enterobacterales* express various antigens (e.g., K, O, and H) (31, 32), ECA is unique in that it is restricted to one order and in which it is invariant (Fig. 1A) allowing cross-reactivity among the members of *Enterobacterales* (33).

Calvin M. Kunin and colleagues first discovered ECA in 1962 (33). The discovery of ECA was a result of studying strains of E. coli causing urinary tract infections and observing the reaction between rabbit antisera generated against the strains and 102 homologous and heterologous E. coli strains. The authors used a standard procedure (passive hemagglutination) to detect O-antigen found in the LPS of the E. coli (33). While carrying out these experiments, they realized there was a cross-reacting specificity between the antisera and many strains of E. coli. Although various antisera demonstrated differing reactivities, anti-E. coli O14 sera reacted with a remarkable range of strains: anti-O14 serum had antibodies recognizing an antigen common to various E. coli strains. However, this antigen was not the LPS-attached O-antigen that Kunin and colleagues had been investigating (33). Furthermore, this cross-reacting antigen was also observed in most other enteric bacteria (33, 34). The antigen was, therefore, named enterobacterial common antigen (ECA) (35).

After the discovery of ECA, research was conducted to ascertain the dissemination of the new antigen among species, eventually aided by a monoclonal ECA antibody that enhanced ECA detection (36). ECA is present in wild-type strains of *Enterobacterales* and absent in both other Gram-negative bacteria and Gram-positive bacteria (Table 1). More studies need to be carried out on the unusual presence of enterobacterial common antigen in Aeromonas hydrophila 209A, as it is not present in the other strains belonging to the same species (37) and may be the result of horizontal gene transfer. Few exceptions to the ubiquitous expression of ECA in *Enterobacterales* exist. These species, which appear to have lost ECA expression, are the endosymbiotic members of *Enterobacterales*, which have a reduced genome size due to the loss of many genes rendered unnecessary by their obligate symbiotic life style (38, 39).

**TABLE 1:** Distribution of ECA in Gram-negative bacteria

Family	ECA positive	ECA negative
Enterobacterales (194)		
*Budviciaceae*	*Leminorella*	
	
	*Pragia*	
*Enterobacteriaceae*	*Atlantibacter*	“Candidatus”
	*Buttiauxella*	
	*Cedecea*	
	*Citrobacter*	
	*Cronobacter*	
	*Enterobacter*	
	*Escherichia*	
	*Gibbsiella*	
	*Izhakiella*	
	*Klebsiella*	
	*Kluyvera*	
	*Kosakonia*	
	*Leclercia*	
	*Lelliottia*	
	*Limnobaculum*	
	*Metakosakonia*	
	*Pluralibacter*	
	*Raoultella*	
	*Salmonella*	
	*Shigella*	
	*Shimwellia*	
*Erwiniaceae*	*Erwinia*	*Buchnera, Wigglesworthia*
	*Mixta*	
	*Pantoea*	
	*Tatumella*	
*Hafniaceae*	*Edwardsiella*	
	*Hafnia*	
	*Obesumbacterium*	
*Morganellaceae*	*Arsenophonus*	“*Candidatus* Arsenophonus lipoptenae”
	*Morganella*	
	*Photorhabdus*	
	*Proteus*	
	*Providencia*	
	*Xenorhabdus*	
*Pectobacteriaceae*	*Brenneria*	
	*Dickeya*	
	*Lonsdalea*	
	*Pectobacterium*	
	*Sodalis*	
*Yersiniaceae*	“*Candidatus* Fukatsuia”	
	*Chania*	
	*Rahnella*	
	*Serratia*	Serratia symbiotica
	*Yersinia*	
Unclassified	*Phytobacter*	
	*Plesiomonas*	
*Vibrionales*		
*Vibrionaceae*	Aeromonas hydrophila 209A	*Vibrio*
Other Gram-negative bacteria		
		*Acidiferrobacter*
		*Actinobacillus*
		*Aeromonas*
		*Alcaligenes*
		*Bordetella*
		*Campylobacter*
		*Cardiobacterium*
		*Chromatiaceae*
		*Chromobacterium*
		*Colwellia*
		*Eikenella*
		*Ferrimonas*
		*Flavobacterium*
		*Gardnerella*
		*Haemophilus*
		*Kingella*
		*Moraxella*
		*Moritella*
		*Shewanella*
		*Nitrobacteriaceae*
		*Pasteurella*
		*Pseudomonas*
		*Rhodospirillaceae*

Antigens that are highly variable between strains of bacteria have served as the foundation for serological naming and grouping. For example, the Kauffmann-Perch scheme is used for *Proteus*, while the Kauffmann-White-Le Minor scheme is used for *Salmonella* (40–42). However, the importance of common antigens has often been overlooked. In recent times, the study of these antigens has increased given their potential significance in vaccine development, determination of phylogeny, and diagnosis. Furthermore, the invariance of common antigens suggests that they have important functions that do not allow for variability. ECA is a perfect example of an antigen that has undergone a recent resurgence of research despite its discovery many years ago. In this review, we explore the history of ECA, its interaction with the immune system, its isolation and biosynthesis, and finally its biological significance.

## THE IMMUNOGENICITY OF ECA

### Interactions of ECA with the immune response.

ECA has a complex interaction with the immune response. Initial studies elucidated that, while the antigen occurred across *Enterobacterales*, just a few sera had antibodies to ECA, for example, E. coli O14 (33, 43). Thus, all strains possessed antigenic ECA but very few possessed immunogenic ECA. The variance in immunogenicity of the strains studied could not be accounted for by differences in the amounts of ECA expressed (35, 44, 45). Therefore, something else must differentiate these types of ECAs. The elucidation of this difference came by separating ECA extracts with ethanol, in which LPS is not soluble, exposing a dissimilarity in the immunogenic types: an ethanol-insoluble immunogenic form and an ethanol-soluble nonimmunogenic form. The ethanol-insoluble form is not separable from LPS and signifies the immunogenic form of the enterobacterial common antigen (46). This form of ECA has ECA bound to the LPS core (ECA_LPS_) (Fig. 1B). The ethanol-soluble form of ECA is not associated with LPS and, instead, consists of the ECA polysaccharide chain covalently linked to diacylglycerol through phosphodiester linkage (ECA_PG_) (47). There is a third form of ECA, cyclic ECA (ECA_CYC_); however, this molecule is found in the periplasm and is not exposed to the environment (48, 49).

Still, why some strains made immunogenic ECA_LPS_ while others did not remained unclear, in part because of the classification of the traditional ECA immunogenic strain, E. coli O14, as an O-antigen-positive strain (50). In fact, the strain is an irregular type of the rough R4 strain disguised by the production of K7 capsular antigen (51). In combination with the production of immunogenic ECA_LPS_ by the R1, R4, and K-12 strains with rough LPS (43), this clarified that ECA_LPS_ is produced in significant amounts only by rough strains that do not make O-antigen. In nonimmunogenic strains, including O-antigen-producing smooth strains and rough strains with incomplete LPS cores or mutations in *waaL*, the O-antigen ligase, ethanol-soluble ECA_PG_ is the predominant form of ECA on the cell surface (35). While some early studies suggested purified ECA_PG_ could induce an antibody response (52), this was only true in strains that also produced significant amounts of ECA_LPS_ (53), suggesting that the antibody production may have resulted from contaminating ECA_LPS_.

With our current knowledge of immunology, it can now be appreciated how the differences in structure between ECA_LPS_ and ECA_PG_ would lead to differences in their immunogenicity. Antibody production is not efficiently stimulated without innate immune signaling (54). ECA_LPS_ possesses an intrinsic adjuvant to stimulate antibody production, as LPS is recognized by toll-like receptor 4 (TLR4), leading to the production of proinflammatory cytokines (55). Nevertheless, as a carbohydrate antigen, ECA_LPS_ mainly stimulates the production of IgM low-affinity, high-avidity antibodies (56). In contrast, the production of high-affinity IgG antibodies requires a protein antigen (57). As a proof of concept of the potential immunogenicity of ECA_PG_, purified ECA_PG_ that contains proteins in addition to ECA can generate an immune response (52). Likewise, a conjugate of ECA and tetanus toxoid, a classic adjuvant, produces ECA antibodies mainly of the IgG isoform (56). Many of the initial studies on ECA immunogenicity were carried out with heat-killed bacteria (50). The difference in immunogenicity of ECA_LPS_ and ECA_PG_ is less in live bacteria (50), likely because of the many pathogen-associated molecular patterns (PAMPs) linked with an active infection and an increased production of proinflammatory cytokines (58).

### Prevalence of ECA antibodies.

Many early studies have reported a low titer of ECA antibodies present in human serum (33, 59, 60), with the caveat that these studies were conducted before the availability of an ECA knockout strain and so may report the combined titer of both ECA antibodies and antibodies to protein antigens shared among *Enterobacterales* (e.g., OmpA). These antibodies have been found in both healthy donors and, at higher levels, in patients with chronic urinary tract infections (61). The titers of ECA antibodies present in the blood have been reported to increase with age (62). A maternal vaccination study reported that the cord blood of a child has lower amounts of ECA antibodies than maternal serum, showing some low level of maternal transfer (61). In other mammals such as cats, dogs, horses, pigs, and mice, ECA antibodies have been reported in blood sera with the exception of rabbits, where no ECA antibody is found. The likely reason for this is due to the high colonization of rabbits by Gram-positive bacteria and low prevalence of E. coli (60). Among several strains of mice, C57B1/6HA mice have ample ECA antibody titers after responding to ECA_LPS_ immunogens (63–65).

Estimates of ECA antibody prevalence in various types of infections have indicated variable titers of ECA antibodies (35). In a few diseases such as enteritis, bacteremia, and acute urinary tract infections, low titers of ECA antibodies were detected. However, higher ECA antibody titers were found in shigellosis (61, 66), peritonitis (53, 67), and chronic urinary tract infections (61, 68, 69). Surprisingly consistent levels of ECA antibodies were observed in a rabbit pyelonephritis model immunized by ECA_LPS_, irrespective of the route of infection and diagnosis (70–72).

The presence of ECA antibodies has been detected in human serum after infection from pathogenic bacteria such as E. coli, Yersinia enterocolitica O3 strains (26, 73–75), and in humans suffering from arthritis associated with Proteus mirabilis strains (76). The ECA immunogenicity is mainly due to ECA_LPS_ (26), with exception to the Rc mutant R4/O28 of P. mirabilis in which ECA_PG_ provides immunogenicity. Therefore, the ECA can be used as a tool for serodetection in various infections caused by the members of the *Enterobacterales* order (77).

### Role of ECA antibodies in host-pathogen interactions.

Several studies have attempted to determine whether ECA antibodies play a role, either protective or pathogenic, in various disease contexts. Under experimental conditions, an appreciable amount of ECA antibodies was found in mice immunized by E. coli O-antigen-negative strains and later challenged by pathogenic bacteria (63–65, 78), except in Swiss albino mice (65). However, protection from these immunizations was partial and temporary. The same partial protection was also observed in mice by passive immunization with serum from rabbits inoculated with E. coli O14 (63). Furthermore, a clinical study has demonstrated that passive immunization with a human monoclonal ECA antibody had no protective effect during sepsis caused by *Enterobacterales* (79).

Bridge et al. (80) reported that, in a mouse model of salmonellosis, infection with the Δ*wecA* strain of Salmonella enterica serovar Typhimurium SL1344 via the oral or intraperitoneal route provided cross-protection against this infection by the production of IgG antibodies. In addition, Huang et al. (81) reported cross-protection against heterologous *Salmonella* strains in mice by downregulating the expression of O-antigen (*rmlB* [*rfbB*]) and ECA (*rmlB_ECA_* [*rffG*]) biosynthesis genes, allowing production of protein-recognizing antibodies. These studies suggest that, at least for *Salmonella*, ECA antibodies may not be protective and that ECA may “distract” the immune system from more efficacious targets. It should be mentioned that some studies have correlated the presence of antibodies to *Enterobacterales*, including to ECA, with rheumatoid arthritis (82–84); however, the causal relationships leading to the antibody production remains unclear.

## FORMS AND BIOSYNTHESIS OF ECA

Over the years, several researchers have developed methods for isolating ECA (Table 2). However, the chemical composition of the antigen was initially difficult to identify, due in part to the existence of ECA in three forms (26) (Fig. 1B). These forms are ECA linked to diacylglycerol through phosphodiester linkage (ECA_PG_), LPS-linked ECA (ECA_LPS_), and periplasmic cyclized ECA (ECA_CYC_) (43, 47, 48). Observations made by Kunin, Neter, and Suzuki initially demonstrated that ECA occurred in two forms in the immunogenic E. coli O14. One of the forms was soluble in aqueous ethanol and was able to be separated from the LPS (ECA_PG_), while the other did not dissolve in ethanol and could not be separated from LPS (ECA_LPS_) (35, 37). ECA_PG_ is the predominant surface-exposed form of ECA, while ECA_LPS,_ the immunogenic form that allows antibody production, is predominantly found in rough mutants that do not make O-antigen (35). Later studies identified a third form of ECA, ECA_CYC_ (48), which is now thought to be present in all species that make ECA (48, 85–88). Initial studies done by Männel and Mayer in 1978 (89) reported that ECA consists of *N-*acetyl-d-glucosamine (GlcNAc) and *N-*acetyl-d-mannosaminuronic acid (ManNAcA). But in 1983, another component of ECA, 4-acetamido-4,6-dideoxy-d-galactose (Fuc4NAc), was identified in *Shigella* by Lugowski et al. (56), demonstrating that ECA consists of a trisaccharide repeating unit (Fig. 1A).

**TABLE 2:** Methods used for study of ECA

Methods used^*a*^	Representative species and strain(s)	Type of ECA	Representative reference(s)
Representative purification methods			
ECA_LPS_			
Hot phenol-water extraction (water phase); dialysis; 90% ethanol precipitation (pellet); anion exchange chromatography	Escherichia coli O1, O14, O55; Shigella flexneri	ECA_LPS_	52
LPS extraction and purification for analysis of ECA_LPS_ and other LPS forms	Yersinia enterocolitica O:3; Shigella sonnei phase II	ECA_LPS_ with LPS	141, 195
ECA_PG_			
Lysis in boiling PBS (supernatant); 85% ethanol precipitation (supernatant)	Salmonella enterica serovar Typhimurium, Salmonella choleraesuis, Salmonella enteritidis, S. flexneri, E. coli O111, O55, O6, O75	ECA_PG_	44, 72
Bacteria killed and dried with acetone; room temperature water extraction; picric acid precipitation (supernatant); acetone precipitation (pellet); Sephadex G200 column chromatography; preparative gel electrophoresis	*Salmonella typhosa* O901	ECA_PG_	196
Hot phenol-water extraction (water phase); phenol-chloroform-petroleum ether extraction (phenol phase); ultracentrifugation (supernatant); anion exchange chromatography	*S.* Montevideo SH94, *S.* Typhimurium, S. sonnei phase I, Plesiomonas shigelloides	ECA_PG_	81, 85, 89, 149, 197
ECA_CYC_			
Sonication in EDTA and lysozyme; boiling water extraction; 85% ethanol precipitation (supernatant); acetone precipitation; column chromatography on silica gel and Sephadex LH-20	S. sonnei	ECA_CYC_	48, 56, 140
Cold trichloroacetic acid extraction; Sephadex G-50 chromatography; anion exchange chromatography	Yersinia pestis EV	ECA_CYC_	86
Sonication in MgSO_4_; ultracentrifugation (supernatant); 75% ethanol precipitation (supernatant); drying and resuspension in ddH_2_O; desalting with ZipTip_C18_	E. coli K-12	ECA_CYC_	88, 111, 145
Hot phenol-water extraction (water phase); DNase, RNase, and protease treatment; ultracentrifugation (supernatant); size exclusion chromatography; Biogel P-100 chromatography	E. coli O157:H^−^	ECA_CYC_ (no *O*-acetylation)	198
Common detection methods			
Passive HA; detection of antigen (whole cell, cell lysates, purified) by coating erythrocytes and assaying agglutination caused by reacting antibodies	E. coli O6, O75, OS:K27, K-12; *S.* Typhimurium TV149 (Ra); *S.* Montevideo SH94 (S); S. sonnei; P. shigelloides; Plesiomonas rettgeri	ECA_LPS_; ECA_PG_; ECA_CYC_; *O-*acetylation required for strong reactivity of serum with ECA	72, 85, 88, 89, 140, 197, 199
HA inhibition; detection by supernatant antigen prevention of agglutination of antigen-coated erythrocytes in the presence of antigen-specific antibody	E. coli O14, O6, O75, K-12; *S.* Typhimurium TV149 (Ra); *S.* Montevideo SH94 (S); S. sonnei	ECA_LPS_; ECA_PG_; ECA_CYC_; *O*-acetylation required for strong reactivity of serum with ECA	52, 72, 89, 111, 140, 199, 200
Immunodiffusion precipitation; identifies antigens after gel electrophoresis through precipitation caused by reaction with antibodies	*S. typhosa* O901; *S.* Montevideo SH94 (S); S. sonnei	ECA_LPS_; ECA_PG_	89, 140, 196
ELISA; quantification of antigens based on their reaction with antibodies	S. sonnei; *S.* Montevideo; P. shigelloides; E. coli OS:K27^−^, K-12; *P. rettgeri*	ECA_LPS_; ECA_PG_	85, 140, 197
Immunoblot; including SDS-PAGE or dot blot followed by immunoblot analysis	*S.* Montevideo SH94; *S.* Typhimurium; E. coli R1, R4, OS:K27^−^, K-12; S. sonnei; P. shigelloides; *P. rettgeri*; Y. enterocolitica O:3	ECA_LPS_; ECA_PG_	81, 141, 145, 197, 201
LC; including liquid-gas chromatography, HPLC, reverse-phase HPLC	S. sonnei; Y. pestis; E. coli K-12	ECA_LPS_; ECA_PG_; ECA_CYC_	56, 86–88, 140
NMR spectroscopy; including ^1^H, ^13^C, and ^31^P	S. sonnei; P. shigelloides; Y. pestis; *S.* Typhimurium LT2; E. coli K-12, O157:H	ECA_LPS_; ECA_PG_; ECA_CYC_	56, 85–87, 149, 195, 198
MS; including gas-LC-MS, gas chromatography-MS, matrix-assisted laser desorption–ionization time of flight	P. shigelloides; E. coli K-12, O157:H^−^; S. sonnei	ECA_LPS_; ECA_PG_; ECA_CYC_	85, 88, 145, 195, 198

a ddH_2_O, double-distilled water; ELISA, enzyme-linked immunosorbent assay; HA, hemagglutination; HPLC, high-pressure liquid chromatography; LC, liquid chromatography; MS, mass spectroscopy; PBS, phosphate-buffered saline.

### Biosynthesis of the ECA polymer.

**FIG 2:**
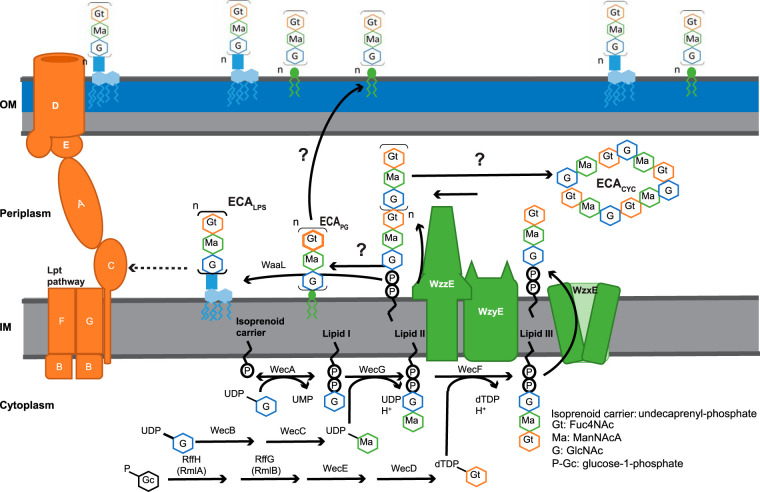
Schematic representation of ECA biogenesis in E. coli. ECA biogenesis begins with synthesis of amino sugars and their attachment to an isoprenoid carrier (Und-P). After a complete subunit is made by series of enzymes namely, WecA, WecB, WecC, WecD, WecE, WecF, WecG, RmlA_ECA_, and RmlB_ECA_, the precursor is flipped across the inner membrane by WzxE, and the subunits are polymerized on the isoprenoid carrier by WzyE with the chain length controlled by WzzE. Three forms of ECA are made from the polymerized subunits: ECA_PG_, attached to diacylglycerol through phosphodiester linkage and surface exposed; ECA_CYC_, which is periplasmic; and ECA_LPS_, attached to LPS and surface exposed. This figure is adapted and modified from Mitchell et al. (145).

The synthesis of ECA is intricate, involving several phases (Fig. 2). The genes necessary for many steps in the synthesis of ECA are located within an operon known as the *wec* operon. The *wec* operon begins at position 85.4 centisomes on the E. coli K-12 chromosome (90), and the functions of each gene of the operon have been analyzed (90–98).

As for many other extracytoplasmic glycans (99), the assembly of the ECA trisaccharide repeat unit is carried out on an isoprenoid lipid carrier, undecaprenyl-phosphate (Und-P), a 55-carbon molecule made of isoprenoid units (93, 100–102). The assembly occurs on the inner side of the plasma membrane (103–107). The first step involves the formation of GlcNAc-pyrophosphoryl-undecaprenol, which is also known as lipid I^ECA^ (108). This step uses UDP-GlcNAc as a substrate to attach GlcNAc-1-phosphate to Und-P and is catalyzed by WecA (93, 101, 102). *In silico* predictions, cysteine accessibility experiments, and fusion-protein expression experiments have demonstrated that WecA has 11 transmembrane segments, with the N terminus in the periplasm and the C terminus in the cytoplasm (109, 110). Furthermore, mutational studies have shown that several conserved aspartate residues in cytoplasmic loops 2 and 3 are important for WecA catalytic activity and divalent cation binding (110). Fluorescence microscopy for green fluorescent protein (GFP)-tagged WecA revealed that it is localized to punctate regions on the cell surface (110). The punctate localization of WecA suggests that ECA and/or O-antigen biosynthesis is localized to discrete cellular regions. The GlcNAc residue of ECA is nonstoichiometrically O-acetylated by WecH, an inner membrane O-acetylase (111).

WecB and WecC are responsible for synthesizing UDP-ManNAcA from UDP-GlcNAc (103, 112, 113). Specifically, WecB (UDP-*N*-acetylglucosamine 2-epimerase) reversibly epimerizes at carbon position 2 between UDP-GlcNAc and UDP-*N*-acetylmannosamine (112, 113). Campbell et al. (114) solved the structure of WecB at a 2.4-Å resolution. This homodimeric enzyme is comprised of two similar sandwich domains with the active site located in the deep cleft at the domain interface. Several basic residues in the active site may have a role in proton transfer at the C-2 position or stabilization of the oxy-carbonium ions in the transition state (114). Residues in the active site have been found to be important for allosteric regulation of WecB as well as for binding and catalysis (115). WecC oxidizes UDP-N-acetylmannosamine in the presence of NAD^+^ to form UDP-ManNAcA (116). The UDP-ManNAcA is the substrate to attach ManNAcA to the lipid I^ECA^, a reaction carried out by WecG (101, 117). This process results in ManNAcA-GlcNAc-pyrophosphoryl-undecaprenol, also known as lipid II^ECA^.

RmlA_ECA_ (RffH), RmlB_ECA_ (RffG), WecE, and WecD are responsible for synthesizing dTDP-Fuc4NAc from glucose-1-phosphate (103, 118, 119). The first reaction, carried out by RmlA_ECA_ (dTDP-glucose pyrophosphorylase 2), forms dTDP-glucose from glucose-1-phosphate, dTTP, and H^+^ (118, 120). Sivaraman et al. (120) solved the RmlA_ECA_ crystal structure in the presence of dTTP and Mg^2+^ ions at a 2.6-Å resolution. This enzyme is tetrameric with each monomer composed of an α/β fold with nucleotide-binding and sugar-binding domains. The active site was identified at the interface of two domains (120).

TDP-glucose acts as a substrate for second enzyme, RmlB_ECA_ (dTDP-glucose 4,6-dehydratase 2). RmlB_ECA_ converts dTDP-glucose to dTDP-4-keto-6-deoxy-d-glucose. This second reaction is a complex reaction that involves dehydrogenation, dehydration, and rereduction in the presence of cofactor NAD^+^ (121). Several active-site residues important for the function of RmlB_ECA_ have been identified based on similarity to UDP-galactose-4-epimerase and mutational analysis (122, 123).

The third reaction is catalyzed by WecE (dTDP-4-dehydro-6-deoxy-d-glucose transaminase), which converts dTDP-4-keto-6-deoxy-d-glucose to dTDP-4-amino-4,6-dideoxy-a-d-galactose (dTDP-Fuc4N) using glutamate as the amino donor (103, 124). A WecE crystal structure has been solved at a resolution of 2.24 Å (125). The structure indicates a homodimeric protein; however, a previous gel filtration experiment suggested a homotetrameric conformation (124). As is common for sugar aminotransferases, the WecE active site contains a conserved lysine that binds the catalytic cofactor, 5′-pyridoxal phosphate, an aspartate important for cofactor activation, and a conserved glutamine (125).

The last reaction is catalyzed by WecD (dTDP-fucosamine acetyltransferase) which uses acetyl coenzyme A (acetyl-CoA) as a cofactor to form dTDP-Fuc4NAc from dTDP-Fuc4N (103, 126). WecD has been crystalized at a resolution of 1.95 Å in its apo form and 1.66 Å in complex with acetyl-CoA (126). The structure shows a dimeric protein with each monomer adopting a GCN5-related *N*-acetyltransferase fold. WecF uses dTDP-Fuc4NAc to transfer Fuc4NAc to lipid II^ECA^, forming Fuc4NAc-ManNAcA-GlcNAc-pyrophosphoryl-undecaprenol (lipid III^ECA^) (103).

The synthesis of lipid III^ECA^ occurs on the inner leaflet of the cytoplasmic membrane facing the cytosol (103); however, polymerization of the trisaccharide repeat unit to form polysaccharide chains occurs on the outer leaflet of the cytoplasmic membrane (127, 128). The flipping of lipid III^ECA^ to the periplasmic face of the membrane is mediated by a “flippase,” WzxE (127, 128). WzxE is a member of the polysaccharide-specific transporter family of proteins, which flip polysaccharides, including some O-antigens and capsular polysaccharides, across the inner membrane (IM) (128, 129). After translocation across the membrane, the ECA chain is polymerized by WzyE. The final chain length of the ECA polymer is determined by WzzE, the chain length regulator (91). These steps result in the formation of an ECA polymer attached to the isoprenoid carrier (Fig. 2).

Several studies have offered insights into the mechanism through which WzzE might control the chain length of ECA. Genetic evidence and cross-linking data support the notion that the flippase, polymerase, and chain length regulator work together as a complex (130, 131). Several structural studies have been performed with WzzE and other members of the class 1 polysaccharide copolymerase family (PCP-1) (132). A crystal structure of the periplasmic domain of WzzE, solved at 2.4 Å, revealed a bell-shaped homo-octameric structure (133); however, reports have suggested various oligomeric states for other PCP-1 family members depending on whether full-length protein was used and the method of analysis (134–137).

This structural inconsistent may be due to the lack of interactions with other complex members (i.e., WzyE). However, recent studies have again suggested octameric structure for both WzzE and WzzB (an E. coli O-antigen PCP-1) (136). The most recent structural data for PCP-1 proteins suggest a mechanism for PCP-1 chain length regulation where polymerization begins when the polymerase and PCP-1 form a complex with the growing polysaccharide chain wrapping either over the surface or through the cavity of the PCP-1 (137). The polymerization could then be terminated either when the PCP-1 and polymerase disassociate (a “stop-watch” mechanism) or when the polysaccharide-binding capacity of the PCP-1 is reached (a “molecular ruler” mechanism) (138–140).

### Synthesis of the three forms of ECA.

The three forms of ECA are made from the ECA polymer. ECA_PG_ is the dominant membrane-associated form of ECA and constitutes about 0.2% of the cellular dry weight of E. coli K-12 (26, 76, 140). This form is present in all *Enterobacterales* (26). The polysaccharide chain is transferred from the isoprenoid carrier to an unidentified lipid to form ECA linked to diacylglycerol through phosphodiester linkage (ECA_PG_) (47). In this molecule, ECA is the head group of the phospholipid (47). The newly synthesized ECA_PG_ is then translocated to the outer membrane (27, 29). The genes and mechanisms involved in the synthesis and translocation of ECA_PG_ remain unknown (87). ECA_LPS_ is synthesized by transferring the linear ECA polysaccharide chain to the core oligosaccharide of LPS (26, 46). This step is catalyzed by WaaL, which is also responsible for attaching O-antigen to the core of LPS (43, 51). However, data suggest that the method for attaching ECA to LPS in Yersinia enterocolitica may be different, allowing ECA and O-antigen to coexist (141). The last form of ECA, ECA_CYC_, is a cyclic molecule consisting of polymerized ECA trisaccharide repeat units, and it is water soluble (26, 48). The ECA_CYC_ is localized in the periplasm (88). This polymer has a variable number of repeat units (4–6) depending on the species (91), and the chain length regulator, WzzE, is necessary for its synthesis (48, 87, 88). A cyclase has not been identified. In-depth structural analysis by crystallography, nuclear magnetic resonance (NMR), and molecular dynamics have suggested that ECA_CYC_ can exist in two three-dimensional conformations (142–144). In contrast to ECA_CYC_, ECA_PG_ and ECA_LPS_ consist of 1 to 14 repeat units, with a modal value of 5 to 7 units in E. coli K-12 (91). In addition, different modal chain lengths have been observed depending on the growth temperature (145).

### Interactions with other biosynthetic pathways.

The use of isoprenoid carriers, such as Und-P, for the synthesis of extracytoplasmic glycans is highly conserved across the domains of life (99, 146, 147). Furthermore, these carriers are often utilized for the synthesis of multiple glycans in the same species. For instance, in E. coli, Und-P is used for the production of ECA, O-antigen, peptidoglycan, and the colanic acid capsule (43, 148–151). Thus, Und-P is a universal lipid carrier required for the synthesis of numerous glycan polymers (152), and this can lead to complex interactions between biosynthetic pathways.

Disruption of one Und-P pathway may lead to indirect consequences on other glycans by altering the amount of Und-P and other precursors available for their synthesis. For example, obstructing the O-antigen pathway in E. coli compromises peptidoglycan biosynthesis by sequestering Und-P (153). In relation to ECA, it was first observed that disruption of later steps in ECA biosynthesis that lead to the accumulation of lipid II^ECA^ causes detergent sensitivity (154) and bile salt sensitivity (155). It was then found that these mutations also lead to the activation of extracytoplasmic stress responses, including Cpx, σ^E^, and Rcs (156–158). Interestingly, in E. coli and Salmonella enterica, these stress responses are only activated with mutations that cause lipid II^ECA^ accumulation (147, 156), but in Serratia marcescens, Rcs activation has been reported even in strains with mutations early in ECA biosynthesis (158). The link of detergent sensitivity and stress response activation to defects in peptidoglycan biosynthesis was conclusively established by the observation that mutations leading to the accumulation of lipid II^ECA^ cause changes in cell shape due to sequestering of Und-P (159). While the accumulation of lipid II^ECA^ is deleterious to the cell, in E. coli, the accumulation of lipid III^ECA^ has been shown to be lethal (88, 128). This has been observed with both loss of WzyE, the ECA polymerase, and loss of the capacity to flip lipid III^ECA^ across the inner membrane (see below) (88, 128). Avoiding lipid III^ECA^ accumulation by disrupting an earlier step in ECA biosynthesis prevents this lethality (88).

The ECA and O-antigen biosynthesis pathways are homologous in *Enterobacterales*. All members of *Enterobacterales* utilize the *wec* locus for the biosynthesis of ECA (102, 160). However, many *Enterobacterales* with GlcNAc as their first O-antigen residue (e.g., Salmonella enterica serovar Minnesota [161], Salmonella enterica serovar Montevideo [162], and E. coli [43]) also utilize *wecA* (*rfe*), the first gene in ECA biosynthesis, for the production of O-antigen chains (163). Therefore, disruptions of *wecA* result in loss of both O-antigen and ECA biosynthesis (43, 164). In addition, the functions of RmlA_ECA_ and RmlB_ECA_, which function in the synthesis of dTDP-Fuc4NAc for ECA biosynthesis, are at least partially redundant with RmlA and RmlB, respectively, two enzymes involved in the synthesis of dTDP-l-rhamnose for O-antigen biosynthesis (118).

The absence of all Wzx family flippases (Wzx_O16_, WzxC, and WzxE) in E. coli K-12 leads to a lethal accumulation of lipid III^ECA^, which can be prevented by the expression of any of the flippases or by prevention of ECA synthesis at an earlier step (88, 128). However, with wild-type expression of *wzx*_O16_ and *wzxC*, deletion of *wzxE* is not lethal, and approximately wild-type levels of ECA_CYC_ are produced (88). These data can be explained by the complex specificity of Wzx family flippases, which has recently been thoroughly reviewed (165). Recent work from Liu et al. (166) suggests “structure-specific triggering,” where flipping is triggered by recognition of specific structural element(s) and other “incorrect” O-antigens may trigger flipping at low frequency when the “correct” substrate is absent (166).

Under normal conditions, the expression of Wzx flippases is low (167), and these flippases show specificity to their canonical substrates (166, 168–172). With various Wzx proteins, the specificity that triggers flipping has been found to be the first sugar residue attached to the Und-PP carrier (168, 169), the presence of terminal side branch residues (170, 171), or the identity or linkage of the terminal residue of the oligosaccharide (166, 171, 172). In the absence of their canonical substrate, the flippases can transport other Und-PP-linked oligosaccharides with various degrees of efficiency depending on their structural similarity, and this transport can be increased by overexpression (166, 170, 171). Thus, in E. coli K-12, which is O-antigen negative but would produce O-antigen with an initial GlcNAc and which does not produce colanic acid unless stressed, the O-antigen and colanic acid Wzx proteins can be utilized with enough efficiency by ECA to prevent a lethal accumulation of lipid III^ECA^ and to produce a measurable accumulation of ECA (88), although the process may be inefficient.

Finally, WaaL, the O-antigen ligase, is responsible for attaching both O-antigen and ECA to LPS (43). Thus, the level of ECA_LPS_ greatly depends on the presence or absence of O-antigen, with very little ECA_LPS_ produced in O-antigen plus strains in E. coli (35). However, Yersinia enterocolitica and Proteus mirabilis produce significant amounts of ECA_LPS_ even in the presence of O-antigen (76, 105). Thus, the interactions between ECA biosynthesis, O-antigen, and peptidoglycan biosynthesis pathways are highly complex. One consequence of these complex interactions is that it confounds the interpretation of high-throughput genetic screens identifying functions of genes in ECA biosynthesis (173–176).

## BIOLOGICAL SIGNIFICANCE OF ECA

The order *Enterobacterales* contains many pathogens important to living organisms, including human beings. Early studies showed that when bacteria are subcultured for prolonged periods under laboratory conditions, the capability to synthesize O-chains diminishes, but there are no effects on the stability of ECA (37). Thus, ECA must have a vital role in *Enterobacterales* (Table 3). However, efforts to clarify the specific biological role of ECA for the enterobacterial cell have failed, partially because of the many interactions between the O-antigen, peptidoglycan (PG), and ECA biosynthesis pathways (43, 144, 156–159). Due to this, very few unambiguously assigned functions have been ascribed to ECA.

**TABLE 3:** Biological significance of ECA in *Enterobacterales*

Function	Type of ECA	Associated gene(s)	Reference(s)
Inhibition of P22 lysis in Salmonella enterica	Complete biosynthesis disruption, possible peptidoglycan stress	*wecB*, *wecC*, *wecD*, *wecE*, *wecG*, and *wzxE*	176
Virulence in *S.* Typhimurium	Loss of all forms of ECA	*wecA*, *wecD*	147, 162
Resistance to toxic molecules (e.g., bile salt, acetic acid, serum, and antibiotics)	Complete loss of ECA, loss O-antigen in some species	*wecA*	150, 160, 178, 181, 182
Resistance to gentamycin	Accumulation of lipid II^ECA^; peptidoglycan stress	*wecE*	179
Resistance to nalidixic and amikacin	Accumulation of lipid III^ECA^; peptidoglycan stress	*wzxE*	180
Maintenance of OM permeability barrier and resistance to detergent and bile salt	ECA_CYC_		111, 145
Proposed regulation of Ca^2+^ ions in the cell	ECA_PG_		52
Maintenance of cell membrane integrity in S. marcescens	Loss of all forms of ECA		183

There has been no difference observed in both the autolysis and the viability in cells having ECA and their counterparts lacking the antigen (177). Barua et al. (178) have shown in the Shiga-toxin-producing E. coli strain O157:H7 that mutants in *wecA* and *wecB* resulting in the loss of ECA and O-antigen or ECA alone, respectively, are sensitive to acetic acid. In addition, there is an increased sensitivity to some antibiotics in mutants lacking ECA, especially aminoglycosides, for example, kanamycin and gentamicin (177). A similar observation was made in E. coli where a *wecE* mutant was found to be sensitive to gentamicin (179). In addition, Girgis et al. (180) observed that a *wzxE* mutant has no phenotype in neutral agar media but, in the presence of nalidixic acid and amikacin, the mutant is sensitive compared to the wild type. A large-scale chemical genetics screen suggested that a *wecA* mutant of E. coli K-12 lacking all ECAs was sensitive to azidothymidine, CHIR-90, minocycline, procaine, puromycin, triclosan, and trimethoprim and resistant to fusidic acid, isoniazid, amdinocillin, vancomycin, and polymyxin (181). A comparison between the ECA_PG_ and the lipopolysaccharide of *Salmonella* Montevideo showed that the ECA_PG_ has a higher Ca^2+^-to-Mg^2+^ ratio than lipopolysaccharide, hence the suggestion that enterobacterial common antigen is important for the supply of calcium ions in the cell (52). Random-transposon mutagenesis experiments performed in Salmonella enterica revealed that disruption of six of the ECA operon genes (*wecB*, *wecC*, *wecD*, *wecE*, *wecG*, and *wzxE*) led to increased speed of lysis by bacteriophage P22 (176). As no effect was observed for disruption of *wecA*, it is possible that this effect is due to the peptidoglycan stress caused by these mutations.

One of the critical roles of ECA is the pathogenicity of *Enterobacterales*, which has been found in some studies. For instance, Salmonella enterica lacking ECA (with mutations in either *wecD* or *wecA*) becomes less virulent and more sensitive to bile salts (147), although it does not use the *wecA* gene for O-antigen biosynthesis, as do many *Enterobacterales*. The phenotype of the *wecD* mutant was more severe than that for the *wecA* mutation, likely due to an accumulation of a Und-P-linked ECA precursor disrupting peptidoglycan synthesis. Gilbreath et al. (182) further validated this result. *In vitro* they found that a *wecA* null mutant of *S.* Typhimurium is deficient in ECA production but fully competent for O-antigen production and lacks stress response activation caused by peptidoglycan biosynthesis disruption. This mutant was highly attenuated in mice, causing a persistent low-level infection that did not kill the mice (182).

Interestingly, mutants defective in ECA biosynthesis trigger Rcs stress response activation in Serratia marcescens regardless of whether peptidoglycan biosynthesis is disrupted, suggesting that ECA may play an especially important role in envelope integrity for this species (150). A role in envelope integrity is also suggested by the overproduction of outer membrane vesicles (OMV) in the absence of ECA in Serratia marcescens, suggesting an instability in the OM (183). In contrast, a screen in E. coli K-12 revealed differences in OMV production in ECA biosynthesis mutants, but these phenotypes varied greatly depending on which gene was mutated, suggesting that the results may be indirect (184). Phan et al. (174) found that seven genes of the ECA operon are essential for serum resistance in E. coli; however, these effects may have been the result of the loss of O-antigen and/or isoprenoid carrier effects.

Recently, we determined that one of the forms of ECA, ECA_CYC_, plays a significant role in the OM permeability barrier (145). We found that loss of ECA_CYC_ disrupts the OM permeability barrier, causing detergent and bile salt sensitivity. Furthermore, we determined that ECA_CYC_ genetically interacts with a protein of unknown function, YhdP, to carry out this activity. When *yhdP* is deleted, ECA_CYC_ takes on aberrant activity that damages the OM, despite greatly lowered ECA_CYC_ levels. Careful screening of different ECA mutants and screening for hallmarks of peptidoglycan stress allowed us to eliminate peptidoglycan stress as a cause of these phenotypes (145).

ECA has been considered as a vaccination against infections stemming from enterobacterial strains due to its prevalence within the order. In a mouse model, salmonellosis leads to the development of ECA antibodies. However, no insight into the role of ECA antibodies in protection from infection was provided (185). Results regarding the protective nature of ECA antibodies have been mixed (63–65, 78, 79, 81). Further investigation into the protective nature of ECA antibodies against *Enterobacterales* species is warranted, as ECA antibodies have the potential to protect against all enterobacterial pathogens. In an ECA vaccine candidate, it would likely be important that the *O*-acetylation of ECA be maintained. *O*-Acetylation of surface-exposed polysaccharide has been shown to be important for the generation of protective antibodies for many pathogens (186–191). Kajimura et al. (111) have reported that the absence of *O*-acetylation in ECA_CYC_ decreases the immunoreactivity of this ECA form toward a rabbit-generated antibody. A recent study has determined that a partially *O*-acetylated polysaccharide may be highly advantageous for vaccine use due to epitope exposure and hydrophobicity profiles (192). In this aspect, ECA may be ideal given its nonstoichiometric acetylation of GlcNAc (111).

## CONCLUDING REMARKS

Since the discovery of ECA in 1962, the work of many investigators has elucidated considerable information about the structure and biogenesis of ECA, and yet our understanding of this fascinating molecule remains incomplete. Whereas many carbohydrate antigens on the bacterial cell surface are highly variable allowing for escape from immune surveillance, ECA remains invariant despite its presence in many pathogenic species. This suggest that the function of ECA must be incredibly important for the cell. Yet, this function is largely unknown. In the past, investigations of the function of ECA have been hampered by the complex genetic and biosynthetic interactions between ECA and other cytoplasmic glycans (i.e., peptidoglycan and O-antigen). Now that these interactions have been characterized, more in-depth studies of ECA functions will be possible.

It is quite likely that the functions of ECA will be found to vary between the types of ECA and to occur on the level of cellular function as well as interaction with the environment. For instance, it has become clear that ECA_CYC_ and ECA_PG_ both play roles in maintaining the OM permeability barrier (145); however, given that antibiotic and detergent susceptibility differs between the loss of these two molecules and the different cellular location of the ECA forms, it is likely that the function of each is distinct. ECA_LPS_, as of yet, has had no cellular function described. While it is clear that ECA is important for pathogenesis, at least in *Salmonella*, it has not yet been determined whether this is due to an alteration of *Salmonella* cellular function or an alteration of *Salmonella*’s interaction with the host.

Nevertheless, from the earliest studies of ECA it has been shown that ECA interacts with the host immune system, leading to the production of broadly cross-reactive antibodies. Yet, it is not known whether these ubiquitous antibodies play a role in the pathogenesis or protection from enterobacterial pathogens. Further evidence of the importance of ECA interactions with the environment can be gleaned from the regulation of ECA expression or chain length by temperature (141, 145, 193). Future investigations into the functions of ECA will lend important insights into the cellular function and host-pathogen interactions of an important group of bacteria.
